# A protocol in action: Recovery approach for patients within high secure care: A 20+ year follow‐up

**DOI:** 10.1002/hsr2.21

**Published:** 2018-02-08

**Authors:** Cheryl Rees, Jamie Pitcairn, Lindsay Thomson

**Affiliations:** ^1^ Division of Psychiatry University of Edinburgh Edinburgh UK; ^2^ The State Hospital and Forensic Managed Care Network Carstairs Scotland UK

**Keywords:** follow‐up, forensic mental health, methodology, recovery

## Abstract

**Objectives:**

A person‐centred approach to recovery is increasingly represented within mainstream mental health literature. Little examination of recovery among forensic mental health patients is evidenced. This study plans to address that insufficiency.

**Methods:**

This protocol paper details a novel approach to exploring recovery among a cohort of 241 patients detained under conditions of high secure care in Scotland during August 1992 to August 1993. Under discussion is the repurposing of previous research to circumnavigate length of inpatient stay commonly associated with forensic mental health care. The methodology adopted, while considering data leakage given the vulnerable participant group, will be discussed.

**Results:**

Repurposing and extending previous research attempts to address the file cabinet effect with 85% of health care research being wasted and future uncertainty regarding research funding in a post‐Brexit era. This is an ongoing study. Ethical, confidentiality, privacy issues, and permissions are considered within the methodology.

**Conclusions:**

Ethical arguments can be made for tracing and attempting contact with vulnerable groups under‐represented in the literature. A well‐considered methodology putting the focus on participant welfare and confidentiality at every step is essential. The reported methodology provides an opportunity to expand and re‐examine previously collected data through a contemporary lens.

## Abstract 2

There is little examination of recovery among forensic mental health patients. This protocol paper details a novel longitudinal approach to exploring recovery among a cohort of 241 patients detained under conditions of high secure care in Scotland during August 1992 to August 1993. Repurposing and extending previous research permits examination through a contemporary lens. Ethical, confidentiality, privacy issues, and permissions are considered within the methodology. Ethical arguments can be made for tracing and attempting contact with underrepresented vulnerable groups.

## 1 INTRODUCTION

During the 2000s, there was a shift in the mental health zeitgeist away from the medical model of symptom reduction to a more holistic and person‐centred approach towards recovery.^1^ Within Scotland, UK, the Mental Health (Care and Treatment) (Scotland) Act 2003^2^ was enacted with the underlying principle of the need to obtain the maximum benefit for patients detained under its provisions, with medical treatment defined in broad terms that include both habilitation and rehabilitation. The person‐centred recovery approach to mental illness focuses on an individual's journey and potential for recovery. This involves developing hope, supportive relationships, coping skills, and a life with meaning.^3^

Through the Mental Health Strategy for Scotland (2017‐2027), The Scottish Government^1^ set out its approach for addressing what has become one of Europe's major health challenges. A commitment has been made to ensuring improved mental health and well‐being services with outcomes delivered for individuals and communities. The expectation of recovery is a theme within the Mental Health Strategy for Scotland (2017) that also includes developing an outcomes approach to include personal, social, and clinical outcomes.

Individuals located within forensic mental health services (which care for people with a mental illness who have been involved with the police, courts, or prison) represent a particularly vulnerable and challenging group with specialist needs. The care cost per week for a long stay psychiatric patient in Scotland is approximately £2000 (2511 USD^4^) whereas for a long stay forensic patient, the cost is approximately £5500 (6905 USD^4^). Patients within forensic mental health services have not only to engage in recovery tasks related to their mental health but also to address the behaviours or offending that has led to their detention within forensic services. As such, the obstacles to be negotiated on the path to recovery will be significantly different for patients within forensic mental health services as opposed to those within mainstream mental health inpatient services.

When considering how to explore recovery within the context of patients passing through high secure care in Scotland, the length of stay associated with forensic inpatient events was a factor in deciding how to supplement existing studies assessing recovery. All patients resident within high secure forensic settings are detained under mental health legislation. Within 2016/2017, patients detained at the State Hospital, Scotland, were spending on average 6 years in high secure care with 65.9% of discharges for that period representing transfers to other National Health Service (NHS) hospitals.^5^

The literature relating to recovery within mental health services is continuing to grow, but there is little representation of forensic patients. The need for research within this area has been previously highlighted.^6^, ^7^ More recently, Clarke et al^8^ and Shepherd et al^9^ have answered this call by publishing meta synthesis of 11 and 5 qualitative studies, respectively, exploring the subjective recovery of forensic mental health patients. Recovery has been most commonly explored qualitatively using a cross‐sectional design. Examination of mortality among forensic patients is one area where longitudinal studies can be found,^10^, ^11^, ^12^ as obtaining information relating to deceased individuals is less bureaucratically intensive^13^ and avoids many thorny ethical issues. We, however, are not aware of any longitudinal studies that have adopted a mixed methods approach, marrying quantitative measures with interviews to explore the subjective experience of recovery, following decade long breaks in contact.

Given the current economic climate of budget constraints and with 85% of health care research reportedly being wasted,^14^ the decision was made to pull some previously collected data out of the filing drawer. Examining it through a contemporary lens cuts costs, breathes new life into previously collected data, and facilitates exploration of issues reliant upon time passed, such as recovery within a forensic mental health context. Uncertainty surrounding Brexit and European funding has also made repurposing and enhancing existing data a more attractive pursuit.

This protocol paper therefore aims to demonstrate how a cohort of patients, first recruited as a descriptive whole population survey^15^ in 1992/1993 then revisited during 2000/2001 to examine risk, offending, and violence^16^ can be repurposed, together with previously collected data, to create a descriptive longitudinal design. This new recovery focused study intends to build on the previous work conducted by the chief investigator to describe in detail the recovery of patients since their detention in high secure care 20+ years ago. As previously mentioned, we have adopted the stance that recovery within the forensic environment is different to that navigated by patients within mainstream mental health services. To that end, we are conceptualising recovery in broad terms to ensure that we examine all facets of the progression from high secure care towards the community. For the purposes of this study, we are examining clinical recovery, functional recovery, social recovery, personal recovery, and offender recovery as described by Drennan and Alred.^17^ Repurposing previous work with this cohort provides a unique opportunity to explore obstructive and supportive factors towards recovery within forensic mental health services.

The overall aims and specific research questions of the recovery approach for patients within a high secure setting: a 20+ year follow‐up are therefore:

### 1.1 Aims

1. To describe the outcomes in terms of the stated recovery approach over a 20+ year period of patients initially detained within a high secure forensic mental health setting.
2. To review the outcomes of patients in terms of the elements of the stated recovery approach; clinical, functional, social, personal, and offender recovery.
3. To compare the outcomes of patients progressing to varying security levels in terms of factors more likely to lead to meaningful recovery.
4. To identify factors associated with readmission to a higher level of security.
5. To determine the current and future needs of high secure care patients and how well they are met within existing resources and services.

### 1.2 Research questions

1. What happens to patients who required high secure care during 1992/1993 over a 20+ year follow‐up period?
2. Are we providing patients with optimal services and resources to promote the various aspects of recovery?

## 2 METHODS

### 2.1 Study setting, eligibility criteria, and recruitment

A cohort of 241 patients (Male N = 213, mean age 36, Female N = 28, mean age 32) resident in the high security State Hospital in Scotland, UK, between August 25, 1992, and August 13, 1993, were identified in the State Hospital Survey.^15^ This cohort was subject to case note data collection (N = 241) and clinical interview (N = 227, 94.2%). All patients had committed acts of serious violence and were admitted from less secure hospitals (due to aggression), from criminal courts (after committing serious offences) or from prison (following deterioration in mental state). Patients were detained under civil or criminal procedures. Table 1 outlines cohort demographics at baseline (1992/1993).

**Table 1:** Demographics N = 241 1992/1993

Demographic	Descriptor	N(%)
Mean age (range)		34.6 (17‐67)
Gender	Male	213 (88.4)
	Female	28 (11.6)
Country of Origin	Scotland	212 (88.0)
	England	17 (7.1)
	Northern Ireland	9 (3.7)
	Ghana	1 (0.4)
	Tunisia	1 (0.4)
	USA	1 (0.4)
Ethnic origin (self‐reported)	Caucasian	238 (98.8)
	Asian	2 (0.8)
	Scottish–Ghanaian	1 (0.4)
Marital status on admission	Single	200 (83.0)
	Married/cohabiting	12 (5.0)
	Divorced/separated	25 (10.4)
	Widowed	4 (1.7)
Employment status when last in community	Employed	56 (23.2)
	Unemployed	185 (76.8)
Best occupational level^18^	I. Professional	3 (1.2)
	II. Managerial/technical	9 (3.7)
	III. Nonmanual	12 (5.0)
	III. Manual	50 (20.7)
	IV. Partly skilled	63 (26.1
	V. Unskilled	61 (25.3)
	Unemployed/ill health	43 (17.8)
Father's socio‐economic status	I. Professional	8 (3.3)
	II. Managerial/technical	18 (7.5)
	III. Nonmanual	9 (3.7)
	III. Manual	40 (16.6)
	IV. Partly skilled	58 (24.1)
	V. Unskilled	29 (12.0)
	Unemployed/ill health/retired	25 (10.4)
	Dead/unknown	54 (22.4)
Admitted from	Court	107 (44.4)
	Prison	48 (19.9)
	Hospital	86 (35.7)
Subject to restrictions (restriction order or direction)^a^		116 (48.1)
Alcohol misuse		117 (48.5)
Drug misuse		113 (46.9)

a Due to having committed serious offences and/or judged to pose a risk of serious harm to others, leave, transfer, or discharge cannot be granted without the permission of the Scottish Government.

This 20+ year follow‐up will, where possible, amalgamate relevant case note and interview data, previously gathered from a specific cohort, for 2 relatively unrelated studies,^15^, ^16^ with new follow‐up information. For deceased/consented participants, case note review will be undertaken to provide an overview of an individual's clinical, functional, and offender recovery for the period from baseline (August 1992 or point of admission to the State Hospital prior to August 1993) to December 2014 (or date of death whichever occurs first). Given the vulnerable participant group and sensitive nature of the research, the study has been designed to engage maximum participation. A single researcher introduces the study and builds rapport, seeks informed consent, and collects participant data. Participants can consent to all or some of the following:

- Participant quantitative and/or qualitative interview,
- Case note review, and/or
- Summary of criminal history since baseline (to aid in examination of offender recovery).

*Inclusion:* This new study intends to follow up the cohort of 241 patients included within the State Hospital patient population survey conducted in 1992/1993.^15^ Only individuals included within the original survey and currently deemed to have the capacity and wellness to consent to participate in the study by their current mental health care team or general practitioner (GP) will be eligible for inclusion.

*Exclusion:* Nonparticipation within the original study sample in 1992/1993 is the main exclusion criteria. This extends to participants from the original cohort who are deemed by their current care team to lack the capacity to consent and/or are not well enough (physically and/or mentally). Those for whom a suitable gatekeeper (mental health care team member/GP) cannot be located are also excluded.

### 2.2 Procedures

The cohort of 241 patients identified within the original State Hospital survey will be traced and their current location found through a combination of means. Although no consent to follow up was obtained at baseline, it was considered by both the South East Scotland Regional Ethics Committee 01 and Public Benefit and Privacy Panel (PBPP) to be in the public interest to examine the pathway through services and personal recovery outcomes for this cohort. We also considered that even if consent to follow up had been requested at baseline, the length of time that has passed since then without contact could invalidate such consent due to changes in the previous participants personal circumstances.

The original paper‐based participant key/cipher that listed patient identifiers, including their unique Scottish Community Health Index (CHI) number (CHI is a population register, which is used in Scotland for health care purposes) against their study index, was still in existence and held securely in a filing cabinet drawer. This key/cipher had been created at baseline (1992/1993) to ensure no duplication of participants and to address ward movement within the hospital. The key/cipher was subsequently used for a first follow‐up (2000/2001) and to inform analysis of the Forensic Network Census data (first collected in 2013, then annually thereafter^19^). The key/cipher was stored as data belonging to the State Hospital, and as such, retention did not breach local retention guidelines. Regarding this current study, ethical approval was sought from the local Caldicott Guardian and research committee who provided ethical approval and use of the key/cipher was brought into line with modern data protection practice.

**Figure 1:**
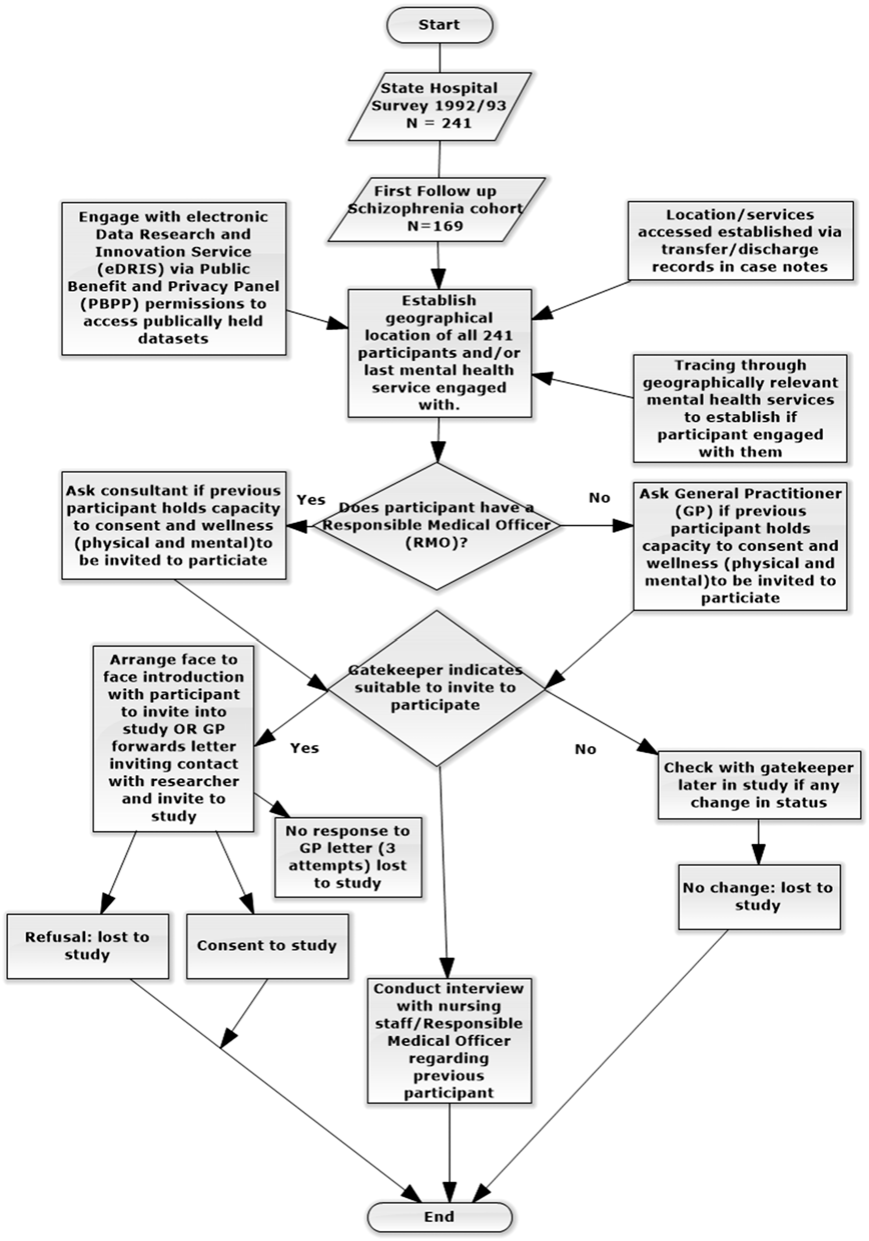
Flowchart outlining ethically approved methodology

Figure 1 outlines the methodology in receipt of ethical approval.

### 2.3 Locating previous participants

Due to the length of time elapsed since initial interview, it is essential that robust recent information be obtained as to the location and status (alive or dead) of participants. Our primary approach is detailed in this section with supplemental strategies examined in ‘Ethical Considerations’ which also outlines how we contemplated the ethical concerns raised by this study.

Support to locate participants was obtained from eDRIS (electronic Data Research and Innovation Service). “The eDRIS service is designed to provide a single point of contact and to assist researchers in study design, approvals and data access in a secure environment.”^20^ Any publically held data can be accessed through eDRIS.

Post ethical approval, we requested scoping information in relation to the 241 previous participants (individual deaths within Scotland, numbers resident within each health board and the number of emigrations from Scotland) from eDRIS through submission of a Confidential Data Release Form (CDRF). As a current/previous care provider, the State Hospital was able to request this information without participant consent.

Following applications to and approval by the Privacy Advisory Committee (PAC), National Caldicott Guardians (NC), and Community Health Index Advisory Group (CHIAG) since amalgamated and refocused as the Public Benefit and Privacy Panel for Health and Social Care (PBPP), more in depth locating information was obtained. Without participant consent, data were supplied by eDRIS for each individual in relation to their most recent Scottish Morbidity Record 04 (SMR04) mental health inpatient event and Scottish Morbidity Record 00 (SMR00) attended mental health outpatient event, the location code of that event and the General Medical Council number for the Responsible Medical Officer (RMO). A gatekeeper approach was adopted whereby each RMO was required to indicate if the previous participant remained within their service, held the capacity to consent to the study, and was physically and/or mentally well enough to be approached for participation. Only following approval from the RMO (or proxy) will an introduction to the participant be arranged.

For individuals no longer in contact with mental health services, their GP will be approached for a gatekeeper decision. If approved, letters of study introduction will be forwarded via their GP practice. If any of the original cohort are traced to the prison service, they will initially be treated as any other previous participant and we will attempt to establish an RMO to seek a gatekeeper decision. If approved for approach, then we will liaise with the prison service to access the individual through the prison health service.

National Health Service Central Registry (NHSCR) will provide notification of those individuals who have died since baseline and continuing notifications of any deaths that occur within the cohort across the United Kingdom for the duration of the study.

Obtaining cohort tracing data through eDRIS has to occur without participant consent as we need to find the previous participants to ask them for informed consent to study interview and/or case note review and/or summary of criminal history again.

**Figure 2:**
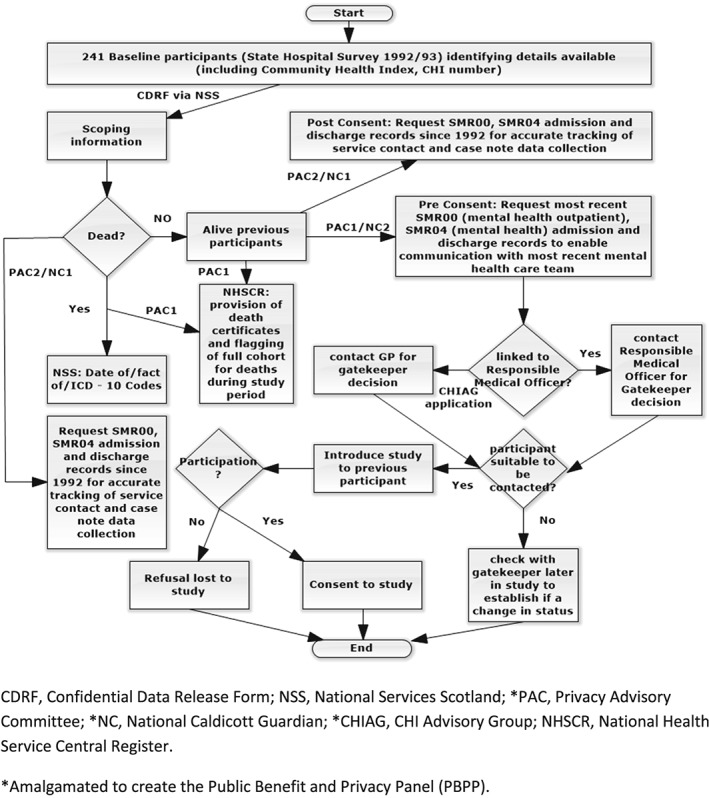
Data flow and permissions (participant tracing, mortality, data, and recruitment)

Figure 2 outlines the organisations engaged with, permissions obtained, and data sourced to trace the previous participants and their current care team, prior to participant consent. For participants who had died since baseline and those that consented to study permissions were requested to enable their route through services from 1992/1993 to be traced and facilitate access to their case notes.

### 2.4 Outcome measures

#### 2.4.1 Data to be repurposed: baseline data

Baseline data for these patients have already been collected from case notes as part of the State Hospital Survey 1992/1993 using a specifically designed data collection tool based on Maden et al^21^ and Johnstone et al.^22^ Data included demographic details; legal status; psychiatric, drug, medical, and forensic history; admission details; social, personal, and family history; diagnoses; and clinical features.

Structured interviews were completed to assess psychopathology and neurological side effects of medication. Tables 2 and 3 detail the tools applied and data collected by participant interview and case note review, respectively, for each stage of the study.

**Table 2:** Tools applied during participant interview

Tools applied during participant interview	Baseline (1992/1993)	First follow‐up (2000/2001)	20+ Year follow up (2015 onwards)	20+ Year follow up recovery aspect
Standardised psychiatric assessment for chronic psychotic disorders (The Manchester)^23^	x	x	x	Clinical
Mania Rating Scale^24^	x			
Depression Rating Scale (MADRS)^25^	x	as subscale of CPRS	as subscale of CPRS	Clinical
Assessment of Involuntary Movements Scale (AIMS)^26^	x	x		
Scale for Targeting Abnormal Kinetic Effects (TAKE)^27^	x	x		
National Adult reading Test^28^	x			
Diagnostic and attainment test^29^	x			
QUICK Test^30^	x	x	x	Functional
Comprehensive Psychopathological Rating Scale (CPRS)^31^		x	x	Clinical
Rating Scale for drug induced akathisia (BARNES)^32^		x		
Brief Psychiatric Rating Scale (BPRS)^33^		x		
Scale for the assessment of negative symptoms (SANS)^34^		x		
Social Dysfunction and Aggression Scale (SDAS)^35^		x	x	Clinical/ functional
Questionnaire about process of recovery (QPR)^36^			x	Personal/social
Semistructured interview based on 7 elements of recovery.			x	Personal/social

**Table 3:** Tools applied/data collected through case note review

Information obtained by Case note review	Baseline (1992/1993)	First follow‐up (2000/2001)	20+ Year follow up (2015 onwards)	20+ Year follow up recovery aspect
St Louis criteria^37^	x			
Present State Examination (PSE) Syndrome checklist^38^	x			
Demographic details	x	x	x	Social
Legal status	x	x	x	Offender
Psychiatric history	x	x	x	Clinical
Drug history	x	x	x	Clinical/functional
Medical and forensic history	x	x	x	Clinical/functional/ offender
Admission details	x			
Social and personal history	x	x	x	Social/functional
Family history	x			
Diagnoses	x	x	x	clinical
Clinical features	x	x	x	clinical
Violence Risk Appraisal Guide (VRAG)^39^		x		
HCR–20^40^ Historical‐10 applied.		x		
The Psychopathy Checklist—Revised (PCL‐R)^41^		x		

#### 2.4.2 Data to be repurposed: first follow‐up

One hundred and sixty‐nine individuals identified from the original cohort who attracted a primary diagnosis of schizophrenia^16^ were followed up during 2000/2001.

Of the 169 individuals, 11 had died by follow‐up. All participants were subject to case note review year by year from 1992 to 2001 or date of death by repeating and extending variables collected at baseline. Interviews were conducted with 142 (89.9%) of the surviving group.

#### 2.4.3 Current study: 20‐year follow‐up

As previously mentioned, to maximise participation and take account of the sensitive nature of the study, individuals can choose the extent to which they wish to consent to participate. Where consented, case note data collection will be supplemented with patient interviews incorporating the previously administered standard psychiatric tools augmented with a questionnaire and semistructured schedule exploring the patient's journey towards social and personal recovery in terms of 7 elements of the recovery approach (hope, secure base, sense of self, supportive relationships, empowerment and inclusion, coping strategies, and meaning and purpose^3^).

Post informed consent or where an individual has died, case note data will be extracted from the notes of all mental health services and/or prisons with which the participant had contact until point of death or December 2014, whichever occurs first. eDRIS will provide information to signpost the mental health services each individual has been engaged with since baseline by extracting data from the Scottish Morbidity Records (SMR). This will ensure that relevant case notes can be located and as full a picture of year by year recovery from 1992/1993 can be established.

The focus of the new aspect of the study, social and personal recovery, will be measured using 2 separate tools: the questionnaire about the process of recovery (QPR^36^), which is a UK developed social/personal recovery tool and a semistructured interview based on 7 elements of recovery.

The QPR was created in conjunction with mainstream service users and originally had 22 items. The psychometric properties of the QPR were assessed in 2014^42^ again with mainstream service users and the interpersonal elements removed due to poor psychometric properties. It was concluded that the tool did not generalise well. The QPR has not been completed by a forensic cohort, and some of the removed items, eg, my recovery has helped challenge other peoples' views about getting better, or I am able to make sense of my distressing experiences, may be more applicable to forensic patients. By administering the 22‐item version, we retain the option to truncate it to the 15‐item version if we wish.

Seven elements of the recovery approach, hope, a secure base, sense of self, supportive relationships, empowerment and inclusion, coping strategies, and meaning and purpose,^3^ have been developed into a semi‐structured interview schedule. Exploration of the recovery elements may highlight themes specific to forensic mental health and will enrich the quantitative findings. Given the nature of the cohort extreme care will be exercised to ensure that no identifying information or quotes are reported, Appendix A contains a truncated version of the full (quantitative and qualitative aspects) of the participant interview.

The full participant interview for this 20+ year follow‐up stage takes approximately 45 minutes to an hour to complete dependent on how much the participant chooses to share with the researcher. Interviews will be completed in a private space within inpatient wards, at outpatient clinics and in participant homes when accompanied by the participant's community psychiatric nurse, when no alternative site is available. For those no longer in contact with community mental health services, they will be interviewed at their GP practice or local community mental health team offices, with any prisoners being interviewed in the prison health centre.

For those within the cohort who remain alive, ethical approval has also been granted for a member of the patient's multidisciplinary team to be interviewed with reference to the patient regardless of the ability of the individual to take part, their refusal or consent to study. This interview was conducted at baseline,^15^ and the tools used are detailed in Table 4.

**Table 4:** Tools applied/data obtained from mental health staff and police

Information obtained by staff interview	Baseline (1992/1993)	First follow‐up (2000/2001)	20+ Year follow up (2015 onwards)	20+ Year follow up recovery aspect
Social Dysfunction and Aggression Scale (SDAS)^35^	x			Clinical/functional
Disability Assessment Schedule (DAS)^43^	x			Functional
Information obtained from Police Service				
Summary of criminal history 1992‐2015			x	Offender

We are seeking to repeat this interview to asses any objective development in functional recovery. We specifically sought ethical approval for this interview in an attempt to obtain data regarding functional recovery for every individual who is alive. We acknowledge that this approval does however raise some concerns where an individual refuses to participate. It is possible that patients could construe the staff interview as being against their wishes and subsequently impact upon the therapeutic relationship. The patient is made aware during study introduction of the involvement of their care team in terms of locating them, the gatekeeper decision and knowledge of their involvement (or refusal) from the perspective of post interview support (if required) and helping them advance their recovery. The staff interview is not explicitly discussed; however, any strong views expressed about their care team involvement would be acknowledged, and in such cases, the staff interview would not be completed. We also acknowledge the expert role of the Regional Ethical Committee who have provided approval for this aspect of the study.

## 3 DATA MANAGEMENT AND ANALYSIS

The data collected through the use of the psychiatric rating tools, criminal records, and case note review (quantitative data) will be coded and entered into Statistical Package for Social Sciences (SPSS^44^ v23), which will allow descriptive data in the form of tables and graphs to be created and the relationships between factors (characteristics) to be explored by regression analysis. Data will be fully anonymised and care taken to ensure that combinations of variables do not lead to the identification of individuals. Data will be processed into individual datasets, eg, patient interview, case note data, and staff interview; however, variables will be merged between datasets to facilitate a deeper exploration of the various aspects of recovery we are examining.

When all data have been processed and checked, it will be assessed using SPSS v23 to ensure that data are missing completely at random. The intention is then to use a process embedded in the software that multiply imputes missing values, each missing data point will then be assigned a value. Scale variables are modelled with a linear regression and categorical variables with logistic regression. Each model uses all other variables as main effects.

### 3.1 Statistical analysis

Statistical analyses will be conducted using SPSS v23. Descriptive statistics will be used (numbers and proportions/percentages) for categorical variables; and means, medians, and ranges for continuous variables.

Bivariate analyses including the chi‐square test will be used when comparing 2 groups on categorical variables (eg, gender) and independent samples *t* test when comparing 2 groups on continuous variables (eg, age). Paired samples *t* test will be used when comparing continuous variables within patients at 2 points in time. Where association between 2 continuous variables is examined, Pearson correlation coefficient will be used. If data are not normally distributed, appropriate nonparametric tests will be used.

With time to event data (eg, time to leave high security, time to reach community, and time to remission of psychosis), survival analysis will be used and Kaplan Meier survival curves will be plotted.

#### 3.1.1 Baseline/follow‐up variable regression model

Logistic regression will be used to examine the independent association between a number of variables (eg, age, previous convictions, and substance misuse) and a yes/no outcome (eg, conviction during follow‐up). Models will be developed for the dependent outcome variable (eg, left high security or convicted during follow‐up) using various baseline and follow‐up factors as independent variables. Variables will be selected based on the literature, bivariate analyses (see above), and clinical relevance. Backward conditional withdrawal of variances will be used identify the variables that best predict the particular outcomes.

#### 3.1.2 Clinical correlates regression model

A regression model will be developed using only variables relating to course of primary diagnosis and co‐morbid conditions as independent variables to look more specifically at the clinical correlates of outcomes.

### 3.2 Qualitative analysis

The data collected to explore personal and social recovery will be the words, phrases, and themes that participants use in response to the questions they will be asked about elements of recovery. An inductive interpretive thematic approach will be taken to the qualitative data captured. Analysis will be supported with NVivo 11^45^ software and analysis undertaken in accordance with Braun and Clarke.^46^ Thematic analysis involves transcribing verbatim the qualitative aspects of the participant interview; reading the transcripts several times to become familiar with the content and noting first thoughts; categories of relevance to the research aims, emergent themes, and commonalities. Categories are then grouped according to consistency in topics and the final themes constructed.

### 3.3 Monitoring

Two members of the research team will undertake reviews of medical records in 5% of cases to explore interrater reliability. For 5% of interviews, the researcher will be joined by an external rater who will score the questionnaires independently. Due to the nature of the participant group who are very familiar with having medical sessions recorded, it is considered effective for data checking to record the full interview rather than just the qualitative sections.

## 4 ETHICAL CONSIDERATIONS

The study was reviewed and approved by the South East Scotland Regional Ethical Committee 01, reference 15/SS/0015. The methodological approach approved is reflected in Figure 1. Discussed below are specific ethical issues arising from the study, incompatibilities between our ethically approved methodology and confidentiality/privacy considerations and suggestions for raising awareness of when an application to the PBPP is appropriate.

This study does represent an ethical minefield, and we carefully considered each and every aspect of the methodology. Beyond general ethical considerations, there are 2 main issues to be addressed. The first regards tracing and contacting patients without consent, we considered 2 aspects in relation to this: (1) Should the study be undertaken at all? We reasoned that not to attempt to explore recovery among forensic mental health patients could be construed as discriminatory given the explosion in recovery research among mainstream services. The same argument also applies to our invitation of patients with a diagnosis of learning disability to participate in the study interview, although researcher discretion is applied once the interview has commenced, as it is for all participants. The interview may be terminated at first sign of distress, truncated or completed over a couple of visits. (2) This study is rooted in the whole population survey^15^ of the State Hospital undertaken in 1992/1993. At that point, the value of developing a longitudinal cohort was not fully appreciated, and even if consent to follow up had been taken at baseline, failure to maintain contact over the intervening decades left us feeling such consent would have been questionable. Having decided that the project should proceed, the focus was then upon conducting it in the most ethically sound manner as possible. As mentioned earlier, to seek consent to participate, we first needed to locate and ask the participants to consent. This raised the second issue: capacity to consent and wellness (mental and physical) to participate among this vulnerable group. To address this concern, a written gatekeeper methodology has been adopted. On tracing a specific individual to an RMO's service, the RMO is written to with an outline of the study requirements for them and their patient. A written response to the question of capacity to consent and general wellness to be invited to participate with reference to the Information Sheets & Consent Forms, Guidance for Researchers and Reviewers, Annex 29^47^ is sought.

Where a participant is no longer in contact with mental health services, their GP will be approached for a gatekeeper decision. Recovery is the focus of this study, and it is deemed appropriate to attempt contact with former participants who are no longer engaged with community mental health teams. We appreciate the pressures on GP's and that they may not have as defined a relationship with the previous participant; however, the expectation is that very few individuals will require a GP gatekeeper decision and it is anticipated that GP's may actually be more cautious than RMO's when considering capacity to consent and general wellness. Only previous participants for whom we can obtain an appropriate gatekeeper decisions regarding capacity/wellness will be included in the study. Due to sensitivities surrounding the high secure care State Hospital and the vulnerability of the patient group, every participant information sheet, consent, letter, and reply slip has been carefully written and approved by the Regional Ethical Committee, National Caldicott Guardian, and PBPP.

This is an ongoing study, and we are actively contacting care teams. For those living within the community, another line of protection has been noted in the form of the individuals' community psychiatric nurse/multidisciplinary care team. A researcher introduction to community‐based individuals is sought from their community team following an appropriate gatekeeper decision. If the community team feel that an approach would be unsettling for their patient despite the RMO giving approval, then this is discussed with the RMO and the decision to approach reversed.

The methodology flowchart, Figure 1, outlines the 3 pronged approach originally mooted and ethically approved to locate the current whereabouts of the previous participants and their current mental health care team. Associated ethical issues for each approach and confidentiality/privacy concerns later realised are discussed.

1. Tracking discharge/transfer notes through the 241 previous participant's paper and electronic records

Despite the shortcomings of the methodology adopted for the first follow‐up study,^16^ it seemed reasonable to include this method as a supplementing strategy for tracing the whereabouts of the previous participants current care team. Despite securing ethical approval, accessing case note data prior to consent is a privacy and confidentiality minefield. National, regional, and local permissions would need to be secured to view records for this purpose, and the local network of confirmations required to source and view case notes makes this nonfeasible. The risk of accidental data disclosure becomes too high, particularly when viewing paper records and previous experience has demonstrated that these records are not always accurate.^16^ It was decided to use the State Hospital Basic Patient Administration System to establish when (and if) previous participants had been transferred out of the State Hospital. This allowed the beginning of an individual's recovery story to be established.

- Tracing through local mental health services

We proposed that if a local area or health board could be established for each previous participant either through discharge/transfer records or from scoping information provided by eDRIS, we could approach local services to enquire if a former participant was engaged with a specific service. As before, this appears a reasonable strategy and again would occur within the context of the NHS and associated confidentiality policies. However, the PAC recommended that such an approach could lead to accidental data leakage. The decision has therefore been made to use contact with local services to confirm information obtained by the third and what has become the primary method for locating previous participants and their gate keepers.

- Supplying unique CHI numbers to the Scottish Electronic Data and Research Innovation Service (eDRIS)

Despite the wealth of information at the disposal of eDRIS for tracing previous participants, the absence of consent to follow up blocked many straightforward routes and PAC and National Caldicott Guardian (NC) applications were required to justify the public benefit of releasing patient identifiable data to support the study. Close work with eDRIS coordinators established which variables and datasets would best support locating each previous participant and their current mental health care team, how the data would flow, be stored and the permissions required to gain access.

Engaging with this process ensured that every piece of correspondence and participant information sheet was not only ethical in approach but was carefully worded to ensure confidentiality and privacy particularly among previous participants who were now living independent lives within the community. Amendments to correspondence were requested by both PAC and NC post ethical approval.

Our research group was aware of the Privacy Advisory Group (PAC), National Caldicott Guardian (NC), and CHIAG requirements from previous work; however, when developing a methodology, the focus is very much on obtaining ethical approval as the first and generally essential requirement. We would like to suggest that privacy and confidentiality considerations be more closely integrated with the ethical approval process at all levels of research from student projects to applications through the online Integrated Research Application System (IRAS). The IRAS could be easily amended to highlight when further approvals or privacy and confidentiality issues require deeper consideration. In this age of “Big Data,” we would welcome a more cohesive approach to ethical, confidentiality, and privacy concerns regarding the use of patient data.

## 5 CONCLUSION

Against the backdrop of budget constraints and the funding uncertainty being ushered in by “Brexit,” this protocol paper seeks to address these issues along with the apparent “waste” of health care research that has plagued the research community for decades. This methodology seeks to encourage other research groups to reassess previously collected data that they may have locked away and breathe new life into it by reviewing such data through the lens of current research priorities. By repurposing data collected from a single cohort for reasons other than recovery, this protocol aims to create a unique, decade's long, descriptive longitudinal study and to collect data both in person and through case note review. The adopted methodology has been outlined along with the permissions process to access data to ensure that all previous participants are located and various aspects of their recovery captured in some way either through participation, deceased case note review, or by interviewing their current mental health care team. Ethical considerations have been explored and suggestions made as to how issues of privacy and confidentiality may be more closely integrated into the ethical approval process.

## CONFLICT OF INTEREST STATEMENT

The authors have no conflicts of interest to declare.

## AUTHOR CONTRIBUTIONS

Conceptualization: Lindsay Thomson, Jamie Pitcairn

Funding Acquisition: Lindsay Thomson, Jamie Pitcairn

Methodology: Cheryl Rees, Lindsay Thomson

Project Administration: Cheryl Rees

Writing ‐ review and editing: Cheryl Rees, Lindsay Thomson, Jamie Pitcairn

Writing ‐ original draft: Cheryl Rees

## APPENDIX A

### PARTICIPANT INTERVIEW SCHEDULE

Section 1.

Comprehensive Psychopathological Rating Scale (CPRS)^29^

Items 1‐40 reported by participant, rated by researcher

Items 41‐65 observed by researcher, rated by researcher

Item 66 Global rating of illness, rated by researcher

Item 67 Assumed reliability of the rating, rated by researcher

Social Dysfunction and Aggression Scale (SDAS)^32^

Items 1‐9 reported by participant, rated by researcher

Item 10 self‐harm, reported by participant, rated by researcher

Section 2.

General questions: incidents, medication,drug/alcohol, staff contact.

In the last month have you been involved in any capacity, in any incidents that have led to police involvement.

**Yes** ‐ *complete table below*.

**No** – *move onto next question*.

**Table:**

Type of Behaviour	Date	Location	Charged?	Convicted?	Disposal	Influence Drug/Alcohol
			Yes/No	Yes/No		Yes/No

In the last month have you been involved in any capacity, in any other incidents involving verbal or physical aggression or violence?

**Yes** ‐ *complete table below*.

**No** – *move onto next question*.

**Table:**

Type of Behaviour	Date	Location	Consequence	Influence Drug/Alcohol
				Yes/No

Medication

Are you currently prescribed any regular medications? **Yes/No** *(if no move onto next question*

What medications are you prescribed? *(Complete table below)*

Are there any medications that you do not take as prescribed? *(Complete table below)*.

*What are the reasons for not taking those medicines?*

**Table:**

medication	dose	route	compliant	Reasons for non‐compliance
			Yes/No	

Alcohol and substance use.

Have you been drinking alcohol over the last month? **Yes/No** *(if no move onto next question)*.

*How do you feel when you stop drinking? (ask questions exploring withdrawal/ dependence symptoms)*.

**Table:**

Number of drinking days	What drank, how much (*to allow unit calculation. E.g. 2 L White cider)*	Withdrawal symptoms	Dependency symptoms

Have you taken any non‐prescribed drugs in the last month? **Yes/No** *(if no move onto next question)*.

**Table:**

Type	Number of days using	Doses per day	Route(s)	Withdrawal symptoms	Dependency symptoms

Contact with services

How often do you see your keyworker?

**Table:**

1	Daily
2	Couple of times a week
3	Weekly
4	Fortnightly
5	Monthly
6	Intermittently
7	Never

How often do you see a psychiatrist?

**Table:**

1	Daily
2	Couple of times a week
3	Weekly
4	Fortnightly
5	Monthly
6	Intermittently
7	Never

Section 3.

QUICK IQ Test^28^

Section 4.

Questionnaire about Process of Recovery (QPR)^33^

**Semi structured interview based on 7 elements of personal recovery (additional probing as required).**

Recovering from an illness or finding ways to change the way you behave is a very personal experience.

Although there are many similarities, no two people recover in the same way.

The journey towards recovery can mean different things to different people.

I would like you to think about the difficulties that brought you to the State Hospital to be cared for at some point during 1992/93. With that in mind I would like to ask you,

**Questions**

What does recovery mean to you?

Where on your recovery journey do you see yourself?

What has been most helpful in your recovery?

What has not been very helpful?

Looking back over the last 20 years since we first spoke to you, have there been any memorable parts of your journey that made you change direction?

*If **Yes**: Do you mind telling me a bit about them?*

*If **No:** Do you feel that you have changed at all since we first spoke to you 20 years ago? In what way? **OR** Why do you think you have found it hard to change?*

Hope

Do you believe that you can recover?

*If **Yes:** When did you first really believe you could recover?*

*What helps you hold onto that belief when you are finding things difficult?*

*If **No:** Have you ever believed you could recover?*

***Yes*** *What happened to make you feel that you couldn't get better?*

*What do you think would help you to find that belief again?*

***OR***

***No*** *What do you think would need to happen to make you believe you could get* *better?*

Secure Base

What do you think of the place where you live?

***If Positive**: do you think it has been helpful in your recovery? Explore*.

***If Negative**: do you think it been unhelpful in your recovery journey? Explore*.

***(If applicable)** What do you think about the staff? Explore*.

Sense of self

It has been 20 or so years since you were first interviewed in the State Hospital…

Has the way you see yourself changed over that time? *Explore*.

What has been the impact of that (change/lack of change) on your recovery journey? *Explore*.

Supportive relationships

(*should know a bit about relationships from the CPRS etc)*

Have you been able to develop friendships where you can be yourself *(not hide your past illness/difficulties)?*

*Can you tell me a bit about them?*

Do you have any other people (*family or professionals)* who give you support?

Do you find yourself having to tell lies about your past? *(Explore ‐ to avoid stigma, fill in blanks in history…)*

Empowerment and Inclusion

Can you make decisions about your recovery?

Does someone else make any decisions?

How does this make you feel?

Do you feel part of a community?

Do you feel part of the wider world?

Coping strategies

Have found the right skills to help you manage your illness?

What works the best for you?

Do you still find yourself using strategies that you know aren't good for you in order to cope? *Explore (self‐harm, destructive, aggressive etc.)*

Meaning and purpose.

What sorts of things make you feel happy with life?

What sorts of things do you do that make you feel like you have achieved something? (work/placement activities/sports other activities)

*If **Negative**: What do you think would allow you to feel happy?*

Post interview the researcher rates observed items of CPRS and completes the Standardised Psychiatric Assessment for Chronic Psychotic Disorders (The Manchester)^21^
